# Time-controlled fasting prevents aging-like mitochondrial changes induced by persistent dietary fat overload in skeletal muscle

**DOI:** 10.1371/journal.pone.0195912

**Published:** 2018-05-09

**Authors:** Daniele Lettieri-Barbato, Stefano Maria Cannata, Viviana Casagrande, Maria Rosa Ciriolo, Katia Aquilano

**Affiliations:** 1 University of Rome Tor Vergata, Dept. Biology, Rome, Italy; 2 University of Rome Tor Vergata, Dept. of Systems Medicine, Rome, Italy; 3 IRCCS San Raffaele La Pisana, Rome, Italy; Universidade do Estado do Rio de Janeiro, BRAZIL

## Abstract

A large body of evidence suggests that persistent dietary fat overload causes mitochondrial dysfunction and systemic metabolic gridlock. Mitochondrial and lipid metabolism in skeletal muscle (SkM) are severely affected upon persistent high fat diet (HFD) leading to premature tissue aging. Here, we designed weekly cycles of fasting (called as time-controlled fasting, TCF) and showed that they were effective in limiting mitochondrial damage and metabolic disturbances induced by HFD. Specifically, TCF was able to prevent the decline of adipose triglyceride lipase (Atgl), maintain efficient mitochondrial respiration in SkM as well as improve blood glucose and lipid profile. Atgl was found to be the mediator of such preventive effects as its downregulation or up-regulation in C2C12 myotubes triggers mitochondrial alteration or protects against the deleterious effects of high fat levels respectively. In conclusion, TCF could represent an effective strategy to limit mitochondrial impairment and metabolic inflexibility that are typically induced by modern western diets or during aging.

## Introduction

Mitochondrial respiratory function progressively declines with age especially in highly oxidative tissues such as skeletal muscle (SkM) resulting in a raise of reactive oxygen species (ROS) emission and oxidative damage [1]. In addition to this intrinsic mitochondrial dysfunction, lifestyle factors may also affect mitochondrial activity [2]. Similarly to aging, persistent dietary fat overload in association with sedentarily is causative of mitochondrial exhaustion and damage driving a systemic metabolic gridlock characterized by high circulating levels of insulin and glucose, and dyslipidaemia [3]. Obesity and type 2 diabetes (T2D) actually depress respiratory function and exacerbate mitochondrial ROS production accelerating SkM dysfunction and aging [4]. Hence targeting mitochondria is regarded as a viable strategy to prevent SkM deterioration during aging [5].

Adipose triglyceride lipase (Atgl) represents the rate-limiting enzyme of cellular triglyceride hydrolysis [6]. Mice lacking Atgl show inadequate metabolic adaptations of the SkM during exercise [7]. By liberating free fatty acids, Atgl is also involved in lipid signalling culminating in the enhancement of mitochondrial oxidative capacity [8, 9]. Atgl progressively declines during aging in SkM and this represents a critical event in the alteration of SkM homeostasis [8, 10]. In particular, Atgl decrease during aging is responsible for reduced expression of antioxidants, increased release of pro-inflammatory cytokines and muscular atrophy [8]. Low Atgl levels also negatively affect muscle mitochondrial oxidative capacity [10].

Dietary interventions such as caloric restriction promote "healthy aging" by inducing modifications in SkM metabolism via the induction of mitochondrial biogenesis and improvement of oxidative metabolism [11]. Fasting is defined as the removal of solid foods as well as of carbohydrate-, protein, or fat-containing beverages over a specific timespan. Physiological fasting lasts about 8–14 hours, mainly including the nocturnal period of the day. Intermittent fasting (IF) diets include eating patterns in which individuals go protracted fasting periods (about 16–48h), with alternating periods of normal food intake [12]. IF is feasible in human and several metabolic health markers are improved both in healthy individuals or in patients with chronic metabolic diseases such as T2D [12, 13]. IF has been shown to prevent or retard the development of age-associated diseases in model organisms and human [12].

Whether IF could prevent the deleterious effects of high fat diet (HFD) on SkM metabolism is still poorly understood. In this work, in order to buffer HFD-induced damage, we aimed at boosting the oxidative capacity of SkM mitochondria by intervening with a fasting-based dietary intervention. In particular, here we tested the effects of fasting cycles, that we termed time-controlled fasting (TCF), on metabolic disturbances and oxidative stress imposed by HFD on SkM. We found that in mice fed with HFD, TCF was able to prevent alteration of systemic metabolism and maintain high Atgl levels and mitochondrial efficiency concomitant to decreased oxidative damage in SkM.

## Materials and methods

### Mice and treatments

Mouse experimentation was conducted in accordance with accepted standard of humane animal care after the approval by relevant local (Institutional Animal Care and Use Committee, Tor Vergata University) and national (Ministry of Health) committees. To calculate the total sample size in the experimental groups, we used the *G-Power analysis software (http://www.gpower.hhu.de). We set the software to F test, one-way ANOVA, and ‘A Priori’ power analysis. Based on our preliminary data, we calculated a difference for glycaemia values between the lowest group (mice subject to 40h fasting) and the highest group (mice fed with HFD for 4 months) of 44 mg/dL over the common standard deviation of 18 and estimated an effect size (f) of 1.04. Under such conditions we calculated n = 5 mice/group (α = 0.05 and β = 0.2) to reject the null hypothesis of no differences.

C57BL/6J young (2 months-age-old) male mice (purchased from Harlan Laboratories S.r.l., Urbino, Italy) were randomly divided in six groups (n = 5 mice/group): 1) mice in normal diet (ND: 3.85 kcal/g among which 10% kcal from fat, 20% from protein and 70% from carbohydrate) for 4 months; 2) mice in high fat diet (HFD: 5.24 kcal/g among which 60% kcal from fat, 20% from protein, and 20% from carbohydrate) for 4 months; 3) mice subjected to alternate fasting (AF: 24h ND, 64h ND, 24h fasting, 80h ND) for 2 weeks; 4) mice subjected to 2 cycles (2 weeks) of time-controlled fasting (TCF: 40h fasting, 24h ND, then 24h fasting and 80h ND each cycle); 5) 2 weeks after ND, mice were fed with HFD in conjunction with ND (HFD/ND: 40h of ND, 24h of HFD, then 24h of ND and 80h HFD) for 4 months; 6) 2 weeks after TCF, mice were fed with HFD in conjunction with TCF (HFD/TCF: 40h fasting, 24h HFD, then 24h fasting and 80h HFD) for 4 months.

ND (#D12450B) and HFD (#D12492) were from Research Diets, INC (New Brunswick, NJ, USA). ND contained 38% sugars, 4% saturated fats, 6% unsaturated fats and casein; HFD contained 20% sugars, 54% saturated fats, 6% unsaturated fats and casein. Unless otherwise stated, mice were maintained at 23.0°C ± 1.0 °C and 55.0% ± 5.0% relative humidity under a 12 h/12 h light/dark cycle (lights on at 6:00 AM, lights off at 6:00 PM).

At the end of treatments (mice in ND), mice were starved for 4h before body weight measurement and blood samples collections for bioclinical analyses. Glucose, cholesterol, alanine transaminase (Alt), aspartate transaminase (Ast) were measured through the automatized KeyLab analyser (BPCBioSed, Italy) using specific colorimetric assay kits (BPCBioSed). Serum β-hydroxybutirate levels were detected by using the Beta HB assay kit (Abcam) according to the manufacturer’s instructions. Successively, mice were sacrificed by cervical dislocation, and fast-twitch skeletal muscle tissues (i.e. gastrocnemius, anterior tibialis), fat depots (epididymal, subcutaneous, brown adipose tissues) and liver were explanted for the scheduled analyses.

### Cells and treatments

The mouse myoblast cell line C2C12 (CRL-1772) was purchased from ATCC (American Type Culture Collection, Bethesda, MD, USA). Cells were cultured in growth medium (GM, Dulbecco modified Eagle medium, DMEM), supplemented with 10% fetal bovine serum, (100 U/100 g/ml) penicillin/streptomycin, 1 mM sodium pyruvate and 10 mM HEPES. Cells were differentiated by replacing GM with differentiation medium (DM: DMEM with 2% horse serum) when reached 95% confluence. After 4 days, once differentiated, C2C12 myotubes were transfected with Atgl or scramble siRNAs (Santa Cruz Biotechnology, Dallas, TX, USA) by using DeliverX Plus kit (Affymetrix, Santa Clara, CA, USA). For Atgl overexpression, differentiated C2C12 myotubes were transfected with the murine Atgl cDNA cloned into pcDNA^™^*4*/*HisMax* mammalian expression vector through Lipofectamine 3000 reagent (ThermoFisher Scientific, Waltham, MA, USA) according to manufacturer’s instruction. Cells were used 24h after transfection.

Differentiated C2C12 cells were treated with 300 μM palmitic acid (PA) for 72h. To check the effectiveness of PA, Oil Red O (ORO) was used to detect intracellular triglycerides content as previously described [14]. Briefly, cells were incubated with ORO and triglycerides quantification was performed after extraction with 4% IGEPAL in isopropanol followed by 550 nm absorbance analysis.

### Bioinformatics analysis

Skeletal muscle microarray data from young and old mice or mice fed with ND and HFD were analysed for transcript expression of Atgl, Glut-4 and other genes implicated in lipid oxidation using gene set enrichment analysis. Specifically, raw microarray data utilized are publically available on GEO under the accession numbers GDS4904, GDS4892 and GDS2612 for young vs old mice comparisons; GDS3078 and GDS3893 for mice fed with ND vs mice fed with HFD comparisons. Gene expression profile analysis was reported as fold change vs young or vs ND group by using GraphPad Prism software.

### Crude mitochondrial fractions

Crude mitochondrial fractions were obtained as previously described with some modifications [15]. Briefly, minced tissues such as gastrocnemius, liver and brown adipose tissue, and cells were homogenized in homogenization buffer (225 mM mannitol, 75 mM sucrose and 30 mM Tris–HCl, pH 7.4, 1 mM EGTA) by glass/Teflon potter. Total homogenates were centrifuged at 800 x g for 5 min at 4 °C for two times. The pellet was discarded whereas supernatant was collected and centrifuged at 17,000 x g for 15 min at 4 °C to obtain crude mitochondrial fractions in the pellet.

### Immunoblotting

Crude mitochondria and total fractions were lysed in RIPA buffer (50 mM Tris-HCl, pH 8.0, 150 mM NaCl, 12 mM deoxycholic acid, 0.5% Nonidet P-40, and protease and phosphatase inhibitors). Five μg proteins were loaded on SDS-PAGE and subjected to Western blotting. Nitrocellulose membranes were incubated with anti-Glut-4 (sc-53566, Santa Cruz Biotechnology), anti-Atgl (SAB2500132, Sigma-Aldrich), anti-ATP5A (ab11020173, Abcam), anti-UQCRC2 (Ab14745, Abcam), anti-Tubulin (T9026, Sigma-Aldrich), anti-vDAC1 (sc-8828, Santa Cruz Biotechnology), anti-TOMM20 (sc-11415, Santa Cruz Biotechnology), anti-Sod2 (sc-30080, Santa Cruz Biotechnology) primary antibodies at 1:1000 dilution. Successively, membranes were incubated with the appropriate horseradish peroxidase-conjugated secondary antibodies. Immunoreactive bands were detected by a FluorChem FC3 System (ProteinSimple, San Jose, CA, USA) after incubation of the membranes with ECL Selected Western Blotting Detection Reagent (GE Healthcare, Pittsburgh, PA, USA). Densitometric analyses of the immunoreactive bands were performed by the FluorChem FC3 Analysis Software.

### RT-qPCR analysis

Total RNA was extracted using TRI Reagent^®^ (Sigma-Aldrich). RNA (3 μg) was retro-transcripted by using M-MLV (Promega, Madison, WI). qPCR was performed in triplicate by using validated qPCR primers (BLAST), Ex TAq qPCR Premix, and the Real-Time PCR LightCycler II (Roche Diagnostics, Indianapolis, IN) as previously described [14]. mRNA levels were normalized to actin mRNA, and the relative mRNA levels were determined through the 2^−ΔΔ*Ct*^ method.

### Mitochondrial membrane potential, citrate synthase activity and protein carbonyls detection

For analysis of mitochondrial membrane potential, C2C12 cells were resuspended in culture medium without serum, whereas crude mitochondria were resuspended in mitochondrial activity buffer (70 mM sucrose, 220 mM mannitol, 2 mM HEPES buffer, 5 mM magnesium chloride, 5 mM potassium phosphate, 1 mM EDTA, 5 mM succinic acid, and 0.1% fatty acid free bovine serum albumin, pH 7.4). C2C12 cells and crude mitochondria were incubated with 250 nM MitoTracker Red CMX ROS for 30 min at 37°C and the analysis was performed through a FACScalibur instrument (Beckton and Dickinson, San Jose`, CA, USA). MitoTracker Red fluorescence intensity was measured in 200,000 mitochondria and 10,000 cells.

Citrate synthase activity was measured by using a Citrate Synthase Activity Assay Kit (Abcam, Cambridge, UK). Carbonylated proteins were detected by using the OxyBlot Protein Oxidation Detection Kit (EMD Millipore).

### Basal oxygen consumption

Basal oxygen consumption (BOC) was determined in whole tissues and crude mitochondria by using the Oxygraph Plus oxygen electrode system (Hansatech Instruments Ltd., Norfolk, UK). Crude mitochondria were resuspended in an appropriate mitochondrial activity buffer (70 mM sucrose, 220 mM mannitol, 2 mM HEPES buffer, 5 mM magnesium chloride, 5 mM potassium phosphate, 1 mM EDTA, 5 mM succinic acid, and 0.1% fatty acid free bovine serum albumin, pH 7.4), whereas tissues were incubated in DMEM without serum. BOC was monitored at 37°C for 6 min and normalized for mitochondrial protein concentration or tissue weight.

### Assay of mitochondrial DNA integrity and mitochondrial mass

Mitochondrial DNA integrity was evaluated according to Gao et al. 2009 [16] with some modifications. Briefly, the reaction mixtures contained 20 ng of total cellular DNA as a template and primers for amplification of the near full-length 15.9-kb fragment of mtDNA (LF, long fragment) were 5-CCT CCC ATT CAT TAT CGC CGC CCT TGC-3 (sense) and 5-GAT GGG GCC GGT AGG TCG ATA AAG GAG-3 (antisense). To produce a 200-bp fragment of mtDNA (SF, short fragment) as control of mtDNA input the primers were 5-CCT CCC ATT CAT TAT CGC CGC CCT TGC-3 (sense) and 5-GTC TGG GTC TCC TAG TAG GTC TGG GAA-3 (antisense). The amplified products were resolved on 0.8% agarose gels (15.9 kb) and 1.2% agarose gels (200 bp) containing 0.5% ethidium bromide. The PCR products were represented as ratio between LF intensity (LFI) and SF intensity (SFI) and expressed as fold change.

For determination of mitochondrial mass, cell and tissue samples were digested with proteinase K and total DNA was extracted through phenol-chloroform and ethanol precipitation method. mtDNA copy number was analysed by qPCR as described by Yatsuga and Suomalainen [17]. 12S rRNA gene primers and actin gene primers were used for mtDNA and nDNA. 12S rRNA levels were normalized to nuclear actin gene, and the relative mtDNA levels were determined through the 2^−ΔΔ*Ct*^ method.

### Energy expenditure

Energy expenditure was measured by indirect calorimetry performed using LabMaster (TSE Systems, Bad Homburg, Germany). All mice were acclimatized for 24h before measurements into individual metabolic chambers at 25 °C, with free access to food (HFD) and water.

Oxygen consumption (VO_2_, expressed as millilitres of O_2_ consumed per kilogram of body weight per minute) and carbon dioxide production (VCO_2_, expressed as milliliters of CO_2_ produced per kilogram of body weight per minute) were recorded every 15 min for 24h, and the data were averaged for each mouse. The respiratory exchange ratio (RER) is defined as VCO_2_/VO_2_ and does not have any units.

### Histochemical analysis and NADH diaphorase activity

To evaluate adipocyte morphology, explanted adipose tissues (epididymal, subcutaneous and brown fat pads) were fixed in Carnoy’s liquid, dehydrated and embedded in paraffin. Successively, paraffin-embedded tissues were cut in 7 μm sections and stained with hematoxylin and eosin (H&E) prior microscope analysis. NADH diaphorase activity has been assayed using a standard methodology on SkM tissue sections as previously described [18].

### Statistical analysis

The results are presented as means ± S.D. Statistical analyses were carried out by using the Student’s *t* test to compare the means of two groups. One-way ANOVA followed by Tukey’s test was used for comparing the means of more than two groups. Differences were considered to be significant at *p* < 0.05.

## Results

### Persistent high fat diet affects glucose disposal and mitochondrial functionality in skeletal muscle

**Fig 1:**
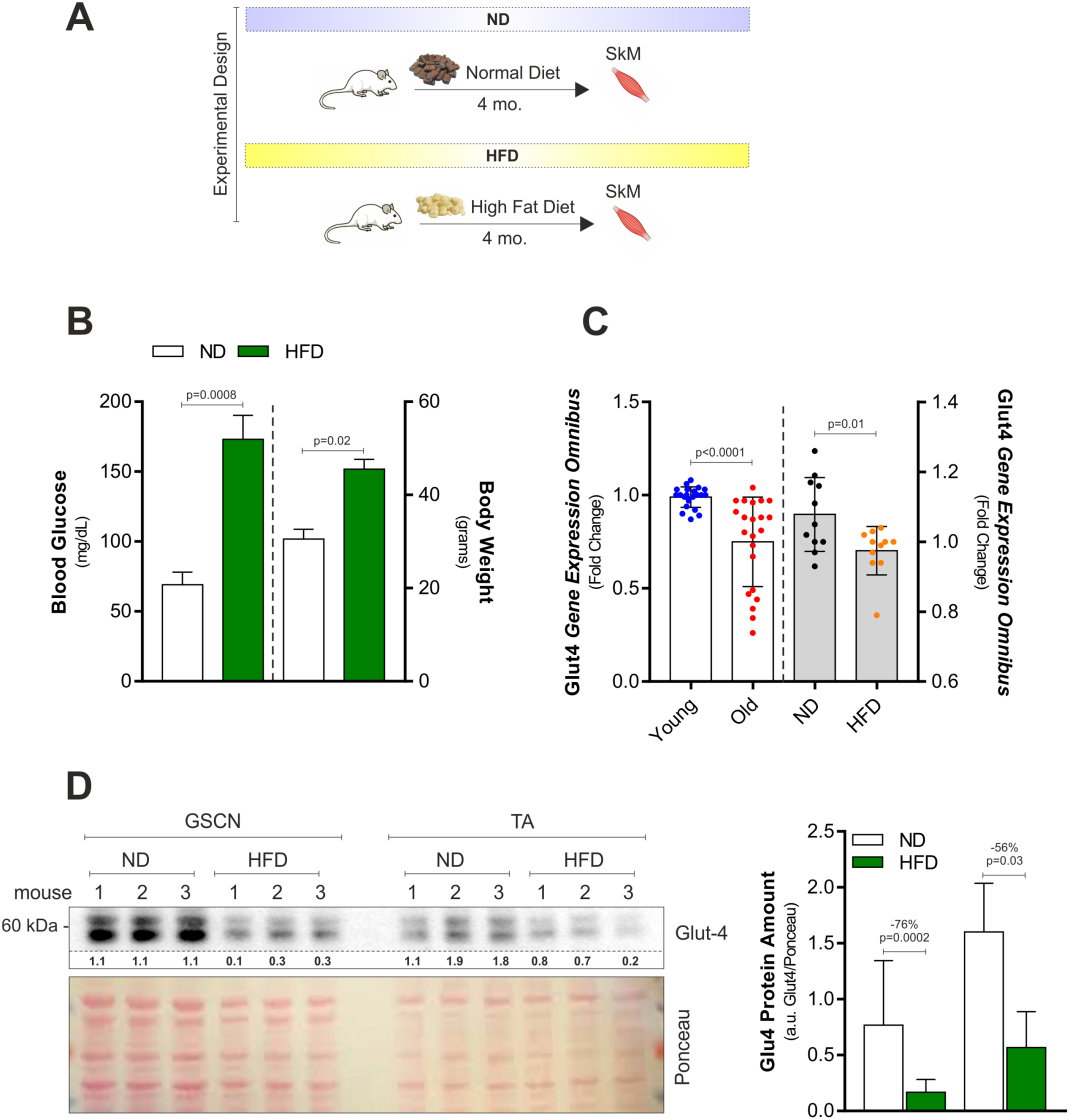
HFD affects Glut-4 and plasma glycaemia in young mice. (A) Schematic representation of the dietary treatment with HFD in C57BL/6J male mice. (B) Blood glucose (*right panel*) and body weight (*right panel*) were measured 4h after fasting in mice fed with normal diet (ND) or high fat diet (HFD). (C) Analyses of data from the publicly available Gene Expression Omnibus (GEO) data set revealed a down-regulation of Glut-4 in SkM of old versus young mice (*left panel*; GDS4904; GDS4892; GDS2612). Analyses of data from the publicly available GEO data set (*right panel*; GDS3078) revealed a down-regulation of Glut-4 in SkM of mice fed with normal diet (ND) or high fat diet (HFD). Gene expression profile was obtained by calculating the fold change of gene expression profile provided by GEO. (D) Western blot analysis of Glut-4 in total homogenates of gastrocnemius (GSCN) and anterior tibialis (TA) of mice fed with ND or HFD. Immunoblots reported are representative of three mice per group out of five giving similar results. Below are reported the densitometric analyses of the immunoreactive bands normalized to Ponceau staining (entire lane). All data are expressed as mean ± S.D. (n = 5 mice/group). Student’s t-test was used to compare ND vs HFD (B, C and D) or young vs old (C).

We fed young C57BL/6J mice with ND or HFD (60% calories from fats) for 4 months (Fig 1A). As reported in Fig 1B, HFD increased body weight (about 48%) and circulating blood glucose levels (about 125%) implicating a reduction of insulin-mediated glucose uptake. Through gene expression data analyses of publically available data (GEO accession number GDS3078), we found that the glucose transporter Glut-4 resulted down-regulated as soon as 28 days in SkM of mice fed with HFD (45% calories from fats) (Fig 1C, grey bars). Similar results were obtained analyzing Glut-4 expression levels in SkM of old mice (GEO accession numbers GDS4904; GDS4892; GDS2612) when compared to young mice (Fig 1C, white bars). To verify whether also in our HFD model glucose uptake was reduced, we determined Glut-4 protein levels. We found that Glut-4 was strongly reduced upon HFD in fast-twitch muscles, i.e. gastrocnemius (GSCN) and anterior tibialis (TA) (Fig 1D).

**Fig 2:**
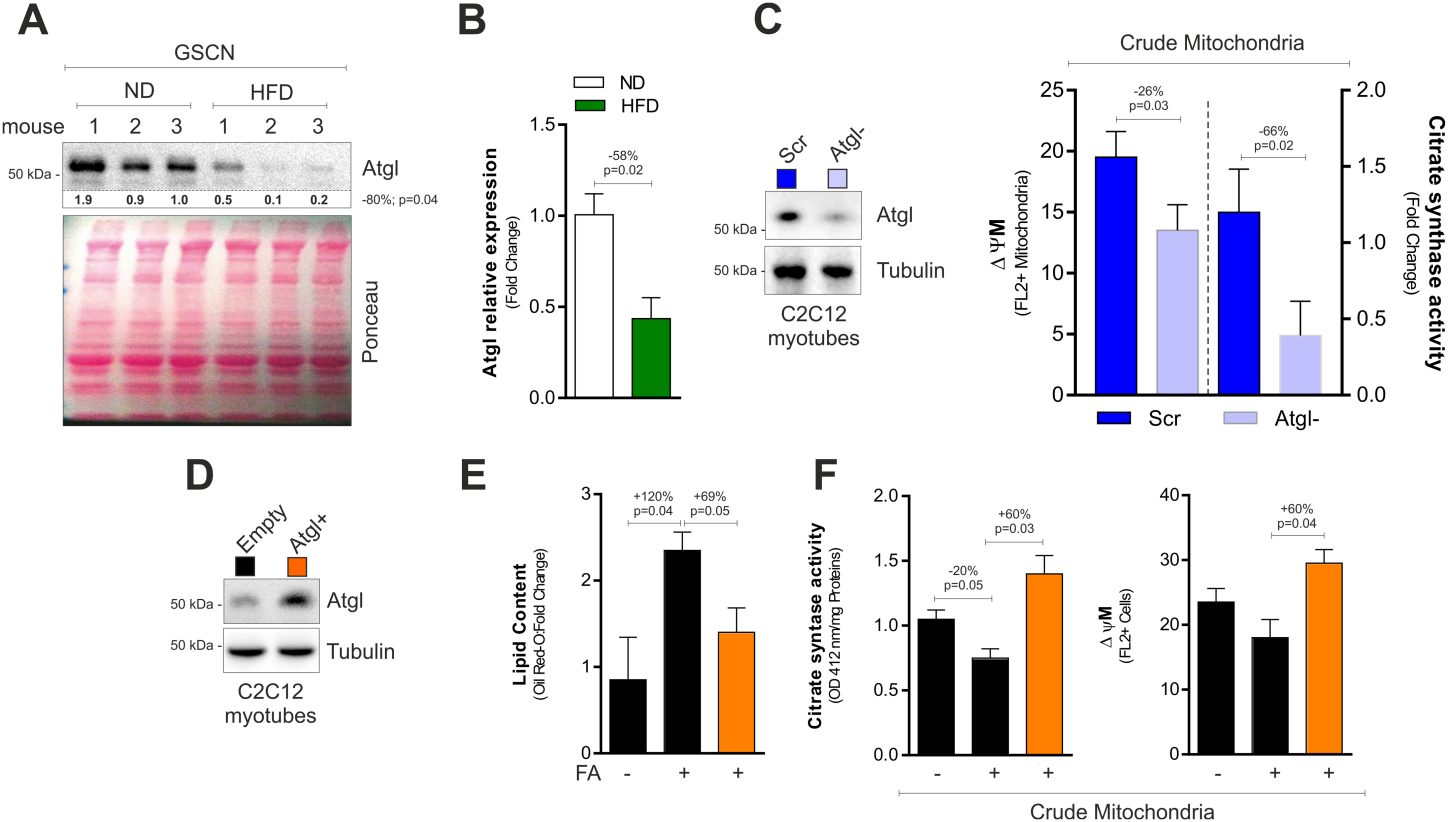
Atgl controls mitochondrial functionality in muscle cells. (A) Western blot analysis of Atgl in total homogenates of GSCN of mice fed with normal diet (ND) or high fat diet (HFD). Immunoblots reported are representative of three mice per group out of five giving similar results. Below are reported the densitometric analyses of the immunoreactive bands normalized to Ponceau staining (entire line). (B) mRNA expression analysis in gastrocnemius (GSCN) upon treatment with HFD. (C) Western blot analysis of Atgl in C2C12 myotubes transfected with a siRNA against Atgl (Atgl-) or with a scramble siRNA (Scr) (*left panel*). Immunoblots reported are representative of one experiment out of three giving similar results. Tubulin was used as loading control. Measurement of mitochondrial membrane potential (ΔΨM) and citrate synthase activity in crude mitochondrial fractions of Atgl- C2C12 myotubes (*right panel*). (D) Lipid content measured by Oil Red O staining (*right panel*) in C2C12 myotubes transfected with the Atgl cDNA (Atgl+) or with empty vector (Empty) and treated with 300 μM palmitic acid (PA) for 72h. Atgl immunoblot (*left panel*) is representative of three independent experiments giving similar results. Tubulin was used as loading control. (E) Measurement of mitochondrial membrane potential (ΔΨM) in crude mitochondrial fractions of Atgl+ C2C12 myotubes. (F) Measurement of citrate synthase activity in crude mitochondrial fractions of Atgl+ C2C12 myotubes. All data are expressed as mean ±S.D. (n = 5 mice/group). In vitro data are representative of at least three independent experiments. Student’s t-test was used for two groups comparisons (A-C). One-way ANOVA analysis followed by Turkey’s test corrections was used for multiple comparisons (D-F).

Fast-twitch fibers result the most affected with aging and are impaired during prolonged exposure to HFD [19, 20]. Thus, we analysed GSCN of HFD-treated mice and detected a significant reduction of Atgl protein (Fig 2A) and mRNA levels (Fig 2B), suggesting an impairment of lipolysis and affected mitochondria. To better understand the role of Atgl in controlling SkM metabolism, we down-regulated it through RNAi in differentiated C2C12 cells. C2C12 cells down-regulating Atgl displayed decreased mitochondrial membrane potential (ΔΨM) and citrate synthase activity (Fig 2C). These results suggested that beside its role in promoting lipolysis, Atgl could be effective in counteracting dysfunction of SkM mitochondria upon HFD. To confirm this assumption, we overexpressed Atgl in differentiated C2C12 cells (Atgl+) and treated them with 300 μM palmitate (PA) for 72h to mimic HFD in mice. As expected, in C2C12 cells with basal Atgl levels, PA promoted intracellular lipids accumulation in association with the drop of ΔΨM and citrate synthase activity (Fig 2D–2F). Notably, Atgl+ cells showed unaffected lipid content and were protected against mitochondrial dysfunction imposed by PA (Fig 2D–2F).

**Fig 3:**
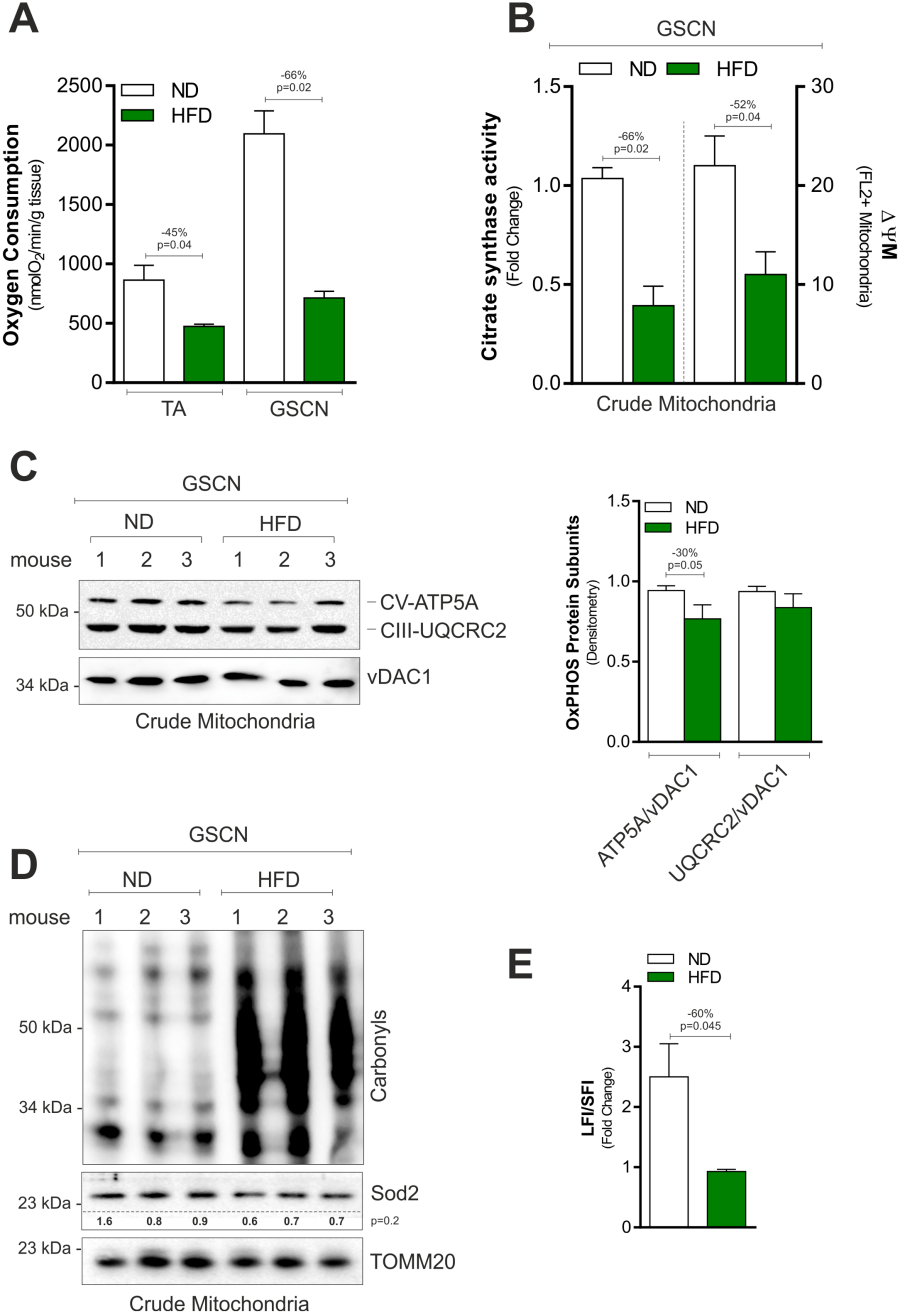
HFD induces mitochondrial damage and dysfunction in SkM. (A) Basal oxygen consumption measured by a polarographic method in gastrocnemius (GSCN) and tibialis anterior muscle (TA) of mice fed with normal diet (ND) or high fat diet (HFD). (B) Citrate synthase activity (*left panel*) and mitochondrial membrane potential (ΔΨM) (*right panel*) measured in crude mitochondria isolated from GSCN of mice fed with ND or HFD. (C) Western blot of CV-ATP5A and CIII-UQRC2 in crude mitochondria isolated from GSCN of mice fed with ND or HFD (*left panel*). Immunoblots reported are representative of three mice per group out of five giving similar results. Densitometric analyses of the immunoreactive bands were performed and normalized to vDAC1 (*right panel*). (D) Western blot analysis of protein carbonyls and Sod2 in crude mitochondria isolated from GSCN of mice fed with ND or HFD. Immunoblots reported are representative of three mice per group out of five giving similar results. Below are reported the densitometric analyses of the immunoreactive bands normalized to TOMM20. (E) mtDNA damage measured by calculating the ratio of long fragment intensity (LFI) and short fragment intensity (SFI) in GSCN of mice fed with ND or HFD. All data are expressed as mean ±S.D. (n = 5 mice/group). Student’s t-test was used to compare ND vs HFD.

To better evaluate the impact of HFD on SkM mitochondria, we initially analyzed the basal oxygen consumption (BOC) in whole SkM tissues (Fig 3A). Unsurprisingly, HFD significantly reduced BOC in TA and GSCN (Fig 3A) and this event was also associated with reduced citrate synthase activity and ΔΨM in crude mitochondrial fractions obtained from GSCN. Moreover, we observed reduced levels of the subunits of the ATP synthase ATP5A (ATP synthase subunit, alpha) (Fig 3C). On the contrary, the subunit of Complex III UQCRC2 (cytochrome b-c1 complex subunit 2) remained unchanged (Fig 3C). In line with this data, we detected increased mitochondrial protein carbonylation (Fig 3D), which represents a marker of oxidative damage. However, this event was not accompanied by the reduction of the main mitochondrial ROS defensive enzyme Sod2 (Fig 3D). mtDNA shows a great vulnerability to oxidative stress [21], hence, we investigated whether mtDNA damage also occurred. To this end, we measured the level of full-length mtDNA and we found that it was lower in the HFD with respect to ND group (Fig 3E), indicating a decreased mtDNA integrity.

### Time controlled fasting up-regulates Atgl dampening mitochondrial dysfunction induced by HFD

**Fig 4:**
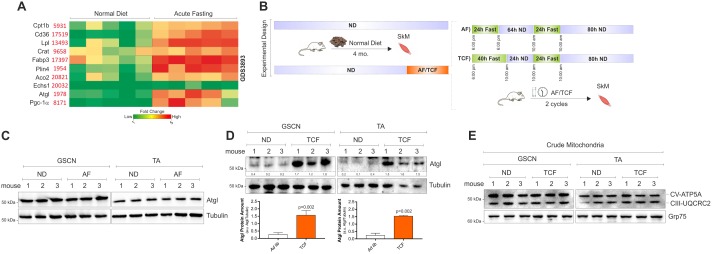
TCF increases Atgl levels in SkM. (A) Heatmap obtained by analysing data from the publicly available Gene Expression Omnibus (GEO) data set (GDS3893) revealed an increase of lipid catabolic genes in SkM of mice subject to fasting versus mice fed with ND. (B) Schematic representation of alternate fasting (AF) and time-controlled fasting (TCF). (C) Western blot analysis of Atgl in total homogenates of GSCN and TA from mice fed with ND or subject to AF for 2 cycles. Immunoblots reported are representative of three mice per group out of five giving similar results. Tubulin was used as loading control. (D) Western blot analysis of Atgl in total homogenates of GSCN and TA from mice fed with ND or subject to TCF for 2 cycles. Immunoblots reported are representative of three mice per group out of five giving similar results. Below are reported the densitometric analyses of the immunoreactive bands normalized to Tubulin. Student’s t-test was used to compare ND vs TCF. (E) Western blot analysis of CV-ATP5A and CIII-UQCRC2 in crude mitochondria isolated from GSCN or TA of mice fed with ND or subject to 2 cycles of TCF. Immunoblots reported are representative of three mice per group out of five giving similar results. Grp75 was used as loading control.

Through gene expression data analyses of publically available data (GEO accession number GDS3893), we found that a wide range of lipid catabolic genes is significantly up-regulated in SkM upon acute fasting including Atgl (Fig 4A). Thus, we tested whether applying non-consecutive days of fasting we were able to maintain Atgl up-regulation. In particular, we carried out an alternate fasting (AF) consisting in two non-consecutive days of fasting (AF: 24h fasting, 64h ND, 24h fasting, 80h ND) in one week for two weeks (Fig 4B). As reported in Fig 4C, Atgl protein levels remained unchanged in SkM of mice treated with AF. We then moved at testing whether, by protracting fasting time, Atgl remained up-regulated. In particular, we designed an intervention consisting in 2 cycles of time-controlled fasting (TCF: 40h fasting, 24h ND, 24h fasting, 80h ND) (Fig 4B). At the end of TCF cycles (two weeks), we observed a significant up-regulation of Atgl protein both in GSCN and TA (Fig 4C). However, no variation was detected in mitochondrial ATP5A and UQCRC2 (Fig 4D).

**Fig 5:**
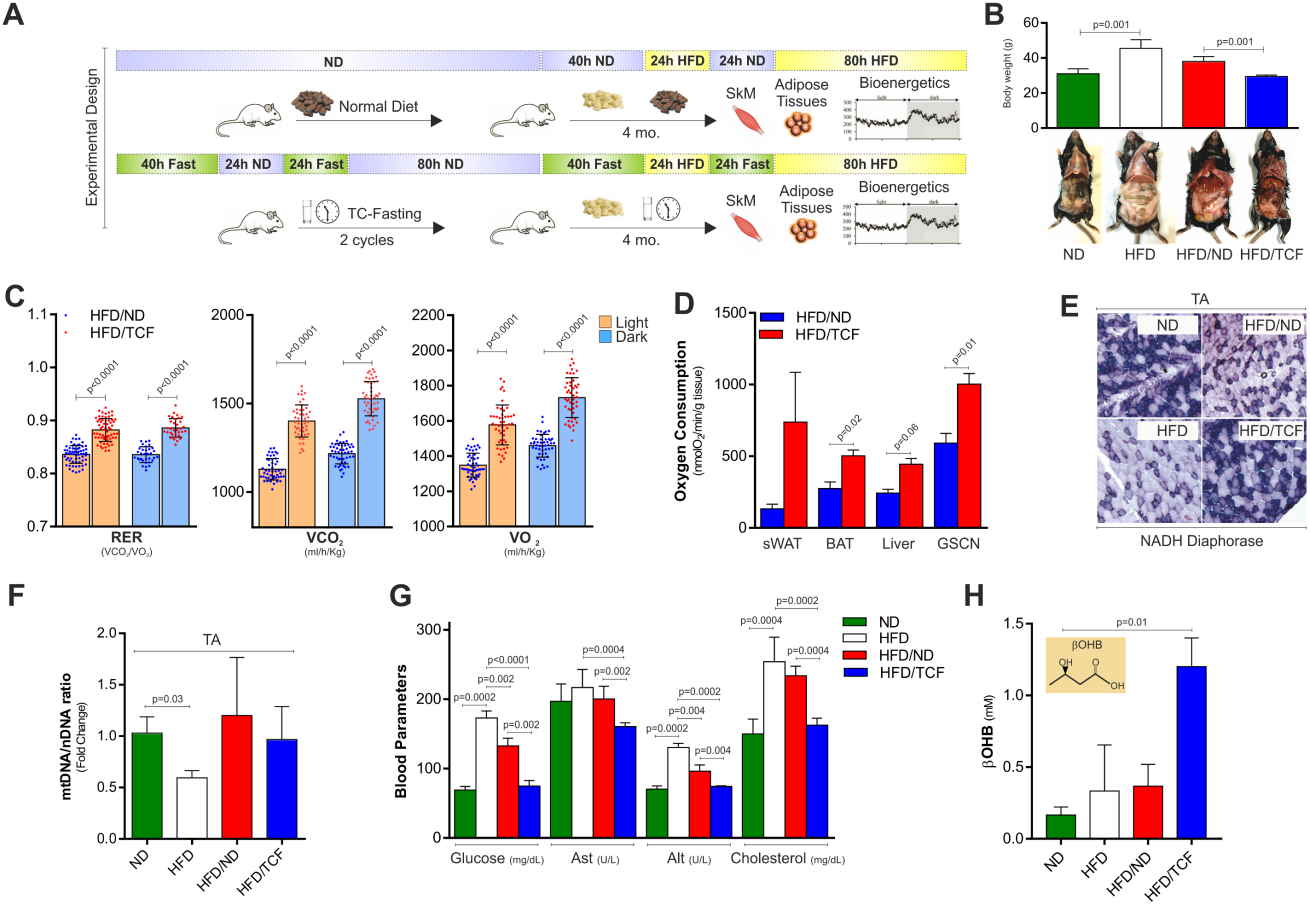
TCF restrains metabolic alterations induced by HFD. (A) Schematic representation of the dietary interventions. (B) Analysis of body weight in mice fed with ND, HFD, alternating HFD with ND (HFD/ND) or TCF (HFD/TCF) (*upper panel*). Representative photograph of mice subject to different dietary treatments (*bottom panel*). (C) RER (VCO_2_/VO_2_), oxygen consumption (VO_2_) and CO_2_ production (VCO_2_) were evaluated by metabolic chamber during nocturnal and diurnal periods in HFD/ND and HFD/TCF mice. During measurement both groups were fed with HFD. (D) Basal oxygen consumption measured by a polarographic method in different tissues of HFD/ND or HFD/TCF mice (sWAT: subcutaneous white adipose tissue, BAT: brown adipose tissue, GSCN: gastrocnemius). (E) Evaluation of Complex I activity in TA by NADH diaphorase staining carried out on tissue slices. Representative images from one mice each group were reported. (F) Mitochondrial mass was evaluated by calculating mtDNA/nDNA through qPCR. (G) Analysis of glucose, Ast (aspartate amino transferase), Alt (alanine amino transferase) and cholesterol levels in the serum. (H) Analysis of beta-hydroxybutyric acid (βOHB) in the serum. All data are expressed as mean ±S.D. (n = 5 mice/group). Student’s t-test was used to compare HFD/ND vs HFD/TCF (C, D). One-way ANOVA analysis followed by Turkey’s test corrections was used for multiple comparisons (B, F, G, H).

In order to test whether TCF was effective in limiting SkM damage induced by HFD, we developed an experimental dietary approach characterized by alternation of TCF with HFD (HFD/TCF) as reported in Fig 5A. HFD was also applied in conjunction with ND (HFD/ND) to test whether a milder reduction in fats and calories could limit HFD-mediated SkM damage as well (Fig 5A). HFD/TCF treatment reduced body weight (Fig 5B) and adipocyte size when compared to HFD/ND (Panel A in S1 Fig). Interestingly, indirect calorimetry revealed a significant increase in the RER (VCO_2_/VO2), oxygen consumption (VO_2_) and CO_2_ production (VCO_2_) in HFD/TCF with respect to HFD/ND group (Fig 5C), in accordance with augmented BOC levels measured in whole tissue lysates (Fig 5D). As reported in Fig 5E, by performing NADH diaphorase staining in TA we detected a decreased Complex I activity in the HFD group that was recovered in HFD/TCF but not in HFD/ND group. However mitochondrial mass evaluated by analyzing mtDNA/nDNA ratio was decreased in HFD and remained affected both in HFD/ND and HFD/TCF (Fig 5F). Similar results were obtained in other tissues wherein no differences in mtDNA/nDNA ratio (Panel B in S1 Fig) as well as in mitochondrial ATP5A and UQRC2 subunits (Panel C in S1 Fig) were observed. Albeit ineffective in preventing mitochondrial mass reduction, these results highlight a strong efficiency of TCF in ameliorating the oxidative efficiency of mitochondria upon HFD. TCF was also effective in improving systemic metabolic profile as demonstrated by reduced blood glucose, cholesterol and Alt (alanine amino transferase) in HFD/TCF with respect to HFD and HFND/ND (Fig 5G). The increase of serum levels of β-hydroxybutyrate only in the HFD/TCF group (Fig 5H) demonstrated that alternating fasting periods with HFD did not affect the ability of fasting to elevate ketone bodies production.

**Fig 6:**
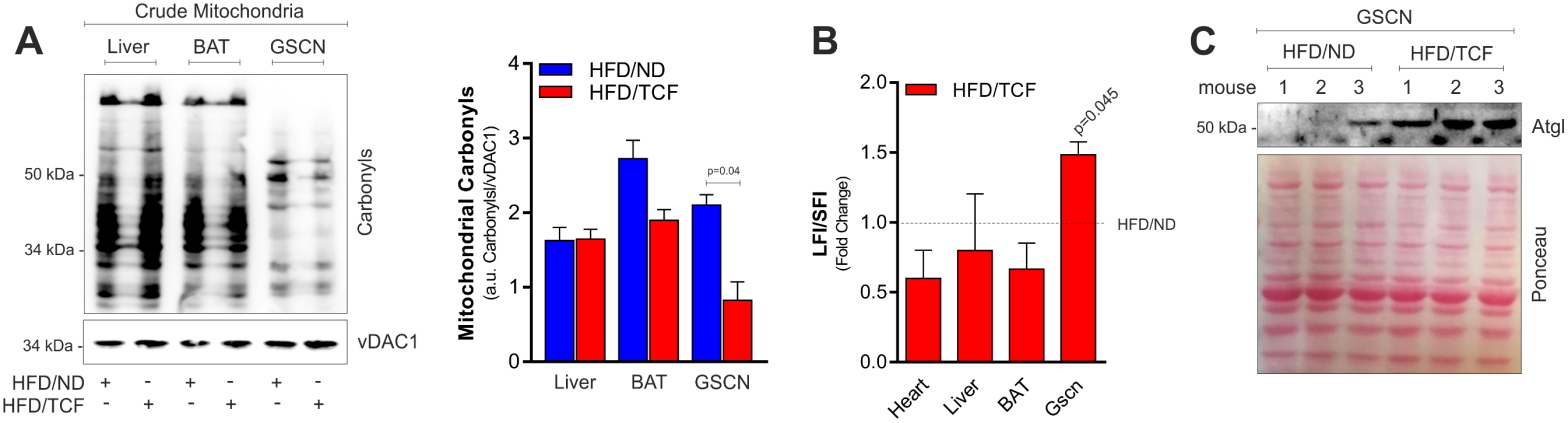
TCF limits mitochondrial damage and increases Atgl levels. (A) Western blot analysis of protein carbonyls in crude mitochondria from different tissues of mice fed with HFD/ND or HFD/TCF. vDAC1 was used as loading control. Immunoblots reported are representative of two mice per group out of five giving similar results. The same vDAC1 was used as loading control in Panel C in S1 Fig. (B) mtDNA damage measured by calculating the ratio of long fragment intensity (LFI) and short fragment intensity (SFI) in different tissues of mice fed with HFD/ND or HFD/TCF. BAT: brown adipose tissue, GSCN: gastrocnemius. (C) Western blot analysis of Atgl in GSCN homogenates of mice fed with HFD/ND or HFD/TCF. Immunoblot reported is representative of three mice per group out of five giving similar results. Ponceau staining was used as loading control. Student’s t-test was used to compare HFD/ND vs HFD/TCF.

Surprisingly, although HFD/TCF increased mitochondrial functionality in SkM, the mitochondrial oxidative damage to proteins and mtDNA was diminished in SkM (Fig 6A and 6B). In particular, we detected a decrease in protein carbonyls (Fig 6A) and maintained mtDNA integrity as assessed by measuring the level of full-length DNA (Fig 6B). Importantly, mitochondria protection observed in SkM was associated with an up-regulation of Atgl (Fig 6C), suggesting that TCF exerts a protective role against HFD via the activation of Atgl-mediated lipid signaling pathway.

## Discussion

Aging is characterized by SkM insulin resistance and wasting as consequence of reductions in mitochondrial oxidative phosphorylation and oxidative damage [22, 23]. Persistent nutrient overload causes severe systemic and chronic metabolic perturbations that in turn lead to premature tissue aging [24]. Herein, we recapitulated the features of SkM aging by feeding healthy young mice with HFD (60% fats). In this model, we observed that persistent HFD affected mitochondrial respiration capacity and promoted a significant lowering of transmembrane potential as well as of ATP5A subunit levels. These results are in line with studies showing that elderly subjects and young insulin resistant offspring from patients with T2D display decreased proton flux through ATP-synthase in SkM [25, 26]. Under nutrient excess, there is a higher flux of electrons to the electron transport chain and this increases the production of ROS that is aggravated especially when ATP synthase activity is also compromised. As consequence, uncontrolled mitochondrial ROS production might induce a significant damage to mitochondria. In line with this evidence, we found a significant oxidative damage to mitochondrial proteins and mtDNA upon HFD.

Healthy mitochondria are able to switch promptly between oxidative metabolism of fatty acids and glucose. When mitochondrial functionality is reduced, the flexibility to adjust substrate oxidation in response to upcoming nutritional changes is disrupted [3]. Mechanistically, the transitions from a fed state back to a fasting state proceed through the relieve of the inhibition of fat oxidation through an intricate mitochondrial metabolic reprogramming. Along with this event, antioxidants are induced and glucose clearance is enhanced [3, 13].

Insulin resistant SkM shows the inflexibility to adapt substrate selection from predominantly fatty acids during fasting to glucose oxidation in the postprandial state [27]. Emerging evidence indicates that boosting mitochondrial function might prevent the decay of body metabolic homeostasis during aging. Caloric restriction and fasting have been claimed to extend longevity through evolutionary conserved mechanisms [28, 29]. Such dietary strategies result beneficial as they improve mitochondrial function in several tissues and promote metabolic health [13, 30]. It was conceivable that alternating protracted fasting periods to HFD may have favorable effects on body and SkM metabolism. It has been reported that in rodents IF slightly reduces body weight, affects mitochondrial bioenergetics and causes redox imbalance in a tissue-specific manner [31, 32]. However, no differences in mitochondrial bioenergetics or redox homeostasis were observed upon this dietary intervention in SkM [31]. Accordingly, here we showed that Atgl levels are not modulated upon AF. Here, we designed a cyclical fasting intervention (TCF) including longer fasting periods (up to 40h). This approach was able to modulate mitochondrial metabolism and maintain high Atgl levels in SkM. Specifically, TCF in conjunction with HFD was proficient in limiting HFD-induced weight gain and protecting against alteration of body metabolic homeostasis. Furthermore, TCF conferred protection against HFD-mediated mitochondrial alteration and oxidative stress. Actually, a recovery of respiration capacity of mitochondria and oxidative fibers amount as well as decreased mitochondrial protein carbonyls and damage to mtDNA in SkM was observed. However, it cannot be excluded that the beneficial effects of TCF may at least in part rely on the prevention of weight gain not to the TCF per se.

Differently to nocturnal fasting (8-14h), TCF also includes diurnal phase characterized by higher metabolic requirement to compensate the muscular activity. These can be achieved by mobilization of fats from adipose depots. Our results suggest that during TCF adipose tissue-released fatty acids represent the main energetic source in SkM. Indeed, increased mitochondrial oxidative capacity was paralleled by a marked decrease of fat mass in HFD/TCF with respect to HFD group.

Importantly, we reported that HFD imposes Atgl decrease in SkM in association with mitochondrial oxidative inefficiency and oxidative stress. Our results are in line with others obtained in rats showing that protracted IF was efficient in activating the oxidation of lipids following HFD with lipid oxidation prevailing over lipogenesis in SkM [33]. Alternating TCF to HFD significantly impedes the decrease of Atgl, supporting the notion that this lipase is directly involved in the protection against metabolic dysfunctions of mitochondria, oxidative damage and accelerated SkM aging. The role of Atgl in buffering HFD-mediated damage was finely demonstrated by experiments carried out in cultured myotubes in which we modulated Atgl protein levels. In particular mitochondrial dysfunction was prevented or exacerbated when Atgl was overexpressed or downregulated respectively. However, other programs that safeguard mitochondria could be operational in response to TCF. Among these SIRT3 activation could be involved, as it is induced upon prolonged fasting and promotes mitochondrial resilience to damage-associated molecular patterns [34].

**Fig 7:**
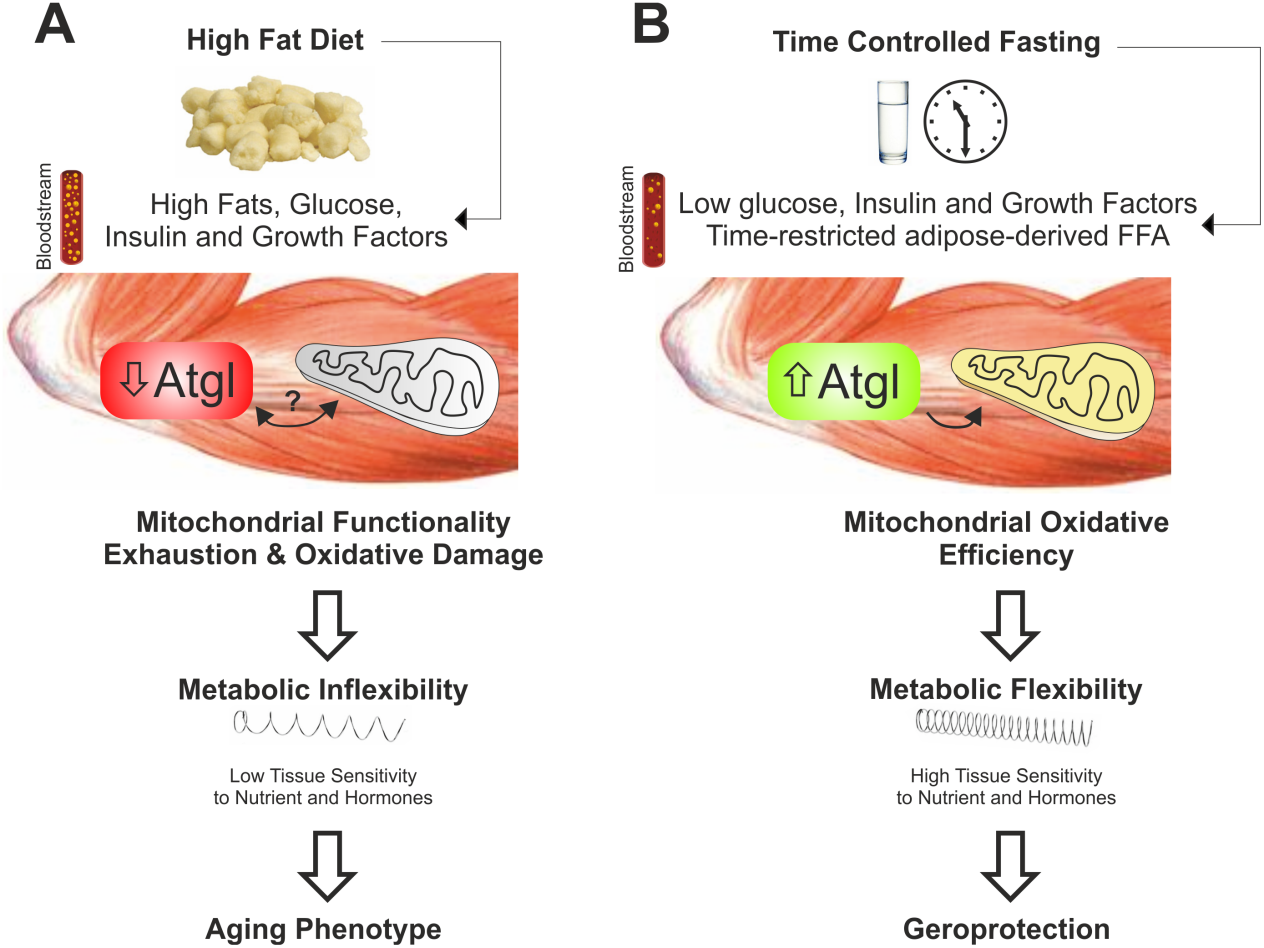
Time-controlled fasting prevents high fat diet-induced metabolic inflexibility in skeletal muscle. (A) High fat diet causes a massive flux of fatty acids into skeletal muscle cells triggering an uncontrolled mitochondrial oxidative metabolism. A persistence of such dietary pattern induces a decrease of Atgl and metabolic inflexibility in skeletal muscle causing elevation of circulating levels of glucose, lipids and insulin. The occurrence of such systemic metabolic gridlock might be the consequence of a protective response (insulin resistance) of metabolically exhausted cells (aging phenotype). (B) Cycles of time-controlled fasting (>24h) boosts mitochondrial oxidative metabolism in skeletal muscle that confers an adaptive stress resistance to nutrient fat overload. Under fasting, the induction of Atgl represents the key molecular event that promotes a controlled release and gradual oxidation of fatty acids into mitochondria. These adaptive responses result in an effective metabolic flexibility preventing high fat diet-induced aging phenotype (geroprotection).

ND in alternation with HFD was only partially effective in protecting against the observed metabolic dysfunctions and this supports the genuine role of TCF in counteracting metabolic and dietary stress and promoting geroprotection. Collectively these findings suggest that alternate cycles of TCF, by maintaining higher Atgl levels in SkM, could avoid metabolic gridlock and inflexibility imposed by HFD and also protect against mitochondrial deterioration and oxidative stress thus retarding SkM aging (Fig 7).

## Supporting information

S1 Fig TCF counteracts HFD-mediated adipose tissue enlargement. (A) H&E staining of adipose tissue depots (sWAT: subcutaneous white adipose tissue, BAT: brown adipose tissue, eWAT: epididymal white adipose tissue) of mice fed with ND, HFD, HFD/ND or HFD/TCF. (B) Mitochondrial mass was evaluated by calculating mtDNA/nDNA through qPCR. (C) Western blot of CV-ATP5A and CIII-UQRC2 in crude mitochondria isolated from liver BAT and GSCN of mice fed with HFD/ND or HFD/TCF. Immunoblots reported are representative of three mice per group out of five giving similar results. vDAC1 was used as loading control.
(TIF)

## Abbreviations

Atgl: adipose triglyceride lipase
ATP5A: ATP synthase subunit, alpha
BOC: basal oxygen consumption
GEO: gene expression omnibus
Glut-4: glucose transporter type 4
GSCN: gastrocnemius
HFD: high fat diet
IF: intermittent fasting
mtDNA: mitochondrial DNA
ND: normal diet
nDNA: nuclear DNA
RER: respiratory exchange ratio
RNAi: RNA interference
ROS: reactive oxygen species
SkM: skeletal muscle
Sod2: manganese superoxide dismutase
T2D: type 2 diabetes
TA: tibialis anterior
TCF: time-controlled fasting
TOMM20: translocase of the outer mitochondrial membrane 20
UQCRC2: cytochrome b-c1 complex subunit 2
vDAC1: voltage-dependent anion channel 1
ΔΨM: mitochondrial transmembrane potential
