# Supplementary figures and images for: Time-controlled fasting prevents aging-like mitochondrial changes induced by persistent dietary fat overload in skeletal muscle
Source: PLoS One. 2018 May 9;13(5):e0195912. doi: 10.1371/journal.pone.0195912 (PMC5942780; doi:10.1371/journal.pone.0195912)

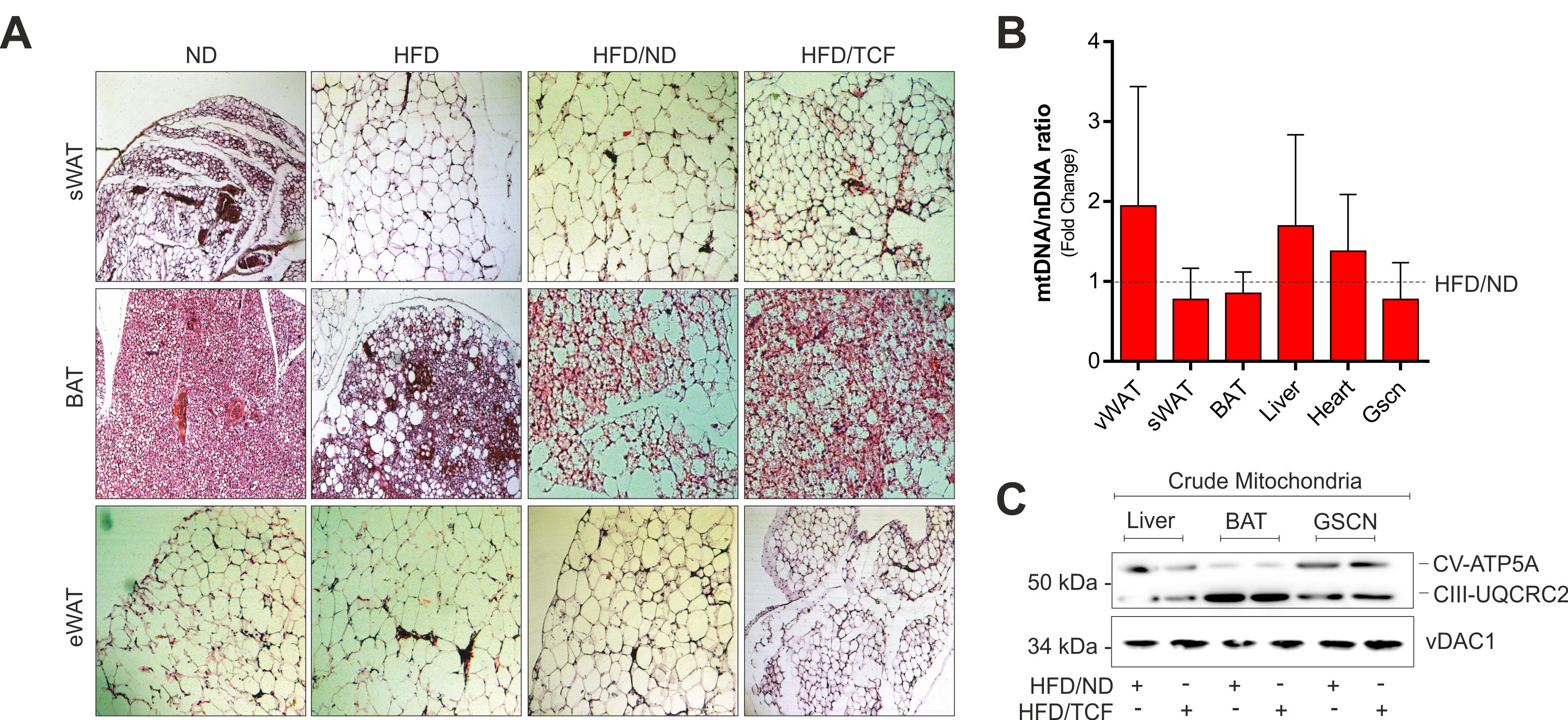

Supplement: S1 Fig — (A) H&E staining of adipose tissue depots (sWAT: subcutaneous white adipose tissue, BAT: brown adipose tissue, eWAT: epididymal white adipose tissue) of mice fed with ND, HFD, HFD/ND or HFD/TCF. (B) Mitochondrial mass was evaluated by calculating mtDNA/nDNA through qPCR. (C) Western blot of CV-ATP5A and CIII-UQRC2 in crude mitochondria isolated from liver BAT and GSCN of mice fed with HFD/ND or HFD/TCF. Immunoblots reported are representative of three mice per group out of five giving similar results. vDAC1 was used as loading control. (TIF) [file pone.0195912.s001.tif]
